# Supplementary material for: The Effect of Visual Capture Towards Subjective Embodiment Within the Full Body Illusion
Source: Sci Rep. 2019 Feb 27;9:2889. doi: 10.1038/s41598-019-39168-4 (PMC6393432; doi:10.1038/s41598-019-39168-4)
Supplement: Supplementary file 1 — Supplementary Material [file 41598_2019_39168_MOESM1_ESM.docx]

**The Effect of Visual Capture Towards Subjective Embodiment Within the Full Body Illusion**

Mark Carey^1*^, Laura Crucianelli^2^, ﻿Catherine Preston^1✝^, and Aikaterini Fotopoulou^2✝^

^1^ Department of Psychology, University of York, York, United Kingdom

^2^ Department of Clinical, Educational and Health Psychology﻿, University College London, United Kingdom

^✝^ These authors have shared senior authorship

1. **Results - Experiment 1**
   1. **Embodiment Questionnaire - Ownership Sub-component Analysis**

Preliminary analysis showed that there was no effect of trial order across visual capture trials, with a Wilcoxon signed-rank test revealing no significant difference in *Ownership* scores between visual capture trial 1 vs. trial 2 (*Z* = - .825, *p* = .409). Therefore, *Ownership* questionnaire scores were collapsed across the two visual capture trials to provide an overall *Ownership* *visual capture* score, per participant.

**1.1.1 Main effect: Visual Capture**

To examine the effects of mere visual capture towards subjective ownership of the mannequin body, we compared *Ownership* scores with *Control* scores in the *embodiment questionnaire*. A Wilcoxon signed-rank test revealed a main effect of visual capture, with significantly higher *Ownership* scores compared with *Control* scores (*Z* = -2.64, *p* = .008, *r* = 42).

**1.1.2 Main effect: Tactile Disruption**

In order to determine whether tactile disruption to participants’ own unseen arm would disrupt subjective ownership, we compared *Ownership* scores between *tactile disruption* and *visual capture* conditions. A Wilcoxon signed-rank test revealed a main effect of condition, in which participants showed significantly lower subjective ownership following *tactile disruption* trials (median = -1.17) compared with *visual capture* trials (median = .33) (Z = -3.88, *p* < .001, *r* = .61).

**1.1.3 Main effect: Tactile Velocity**

Next, we examined whether tactile velocity had an effect in disrupting the subjective ownership towards the mannequin body within *tactile disruption* trials. A Wilcoxon signed-rank test revealed that there was no significant difference in *Ownership* scores between affective and non-affective tactile disruption trials (*Z* = -.309, *p* = .757, *r* = .05), which suggests that interoceptive affective touch did not disrupt visual capture of ownership to a greater degree than exteroceptive, non-affective touch.

- 1. **Embodiment Questionnaire - Location Sub-component Analysis**

Preliminary analysis showed that there was no effect of trial order across visual capture trials, with a Wilcoxon signed-rank test revealing no significant difference in *Location* scores between visual capture trial 1 vs. trial 2 (*Z* = - 1.60, *p* = .111). Therefore, *Location* questionnaire scores were collapsed across the two visual capture trials to provide an overall *visual capture* *Location* score, per participant.

**1.2.1 Main effect: Visual Capture**

To examine the effects of mere visual capture towards subjective ownership of the mannequin body, we compared *Location* scores with *Control* scores in the *embodiment questionnaire*. A Wilcoxon signed-rank test revealed a main effect of visual capture, with significantly higher *Location* scores compared with *Control* scores (*Z* = -5.33, *p* < .001, *r* = 84).

**1.2.2 Main effect: Tactile Disruption**

In order to determine whether tactile disruption to participants’ own unseen arm would disrupt subjective location, we compared *Location* scores between *tactile disruption* and *visual capture* conditions. A Wilcoxon signed-rank test revealed a main effect of condition, in which participants showed significantly lower felt location towards the mannequin following *tactile disruption* trials (median = 1.25) compared with *visual capture* trials (median = 2.00) (Z = -2.59, *p* = .01, *r* = .41).

**1.2.3 Main effect: Tactile Velocity**

Next, we examined whether tactile velocity had an effect in disrupting the subjective location that participants felt within *tactile disruption* trials. A Wilcoxon signed-rank test revealed that there was no significant difference in *Location* scores between affective and non-affective tactile disruption trials (*Z* = -1.04, *p* = .300, *r* = .16), which suggests that interoceptive affective touch did not disrupt felt location to a greater degree than exteroceptive, non-affective touch.

1. **Results - Experiment 2**
   1. **Embodiment Questionnaire - Ownership Sub-component Analysis**

Preliminary analysis showed that there was no effect of trial order across visual capture trials, with a Wilcoxon signed-rank test revealing no significant difference in *Ownership* scores between visual capture trial 1 vs. trial 2 (*Z* = - .651, *p* = .515). Therefore, *Ownership* questionnaire scores were collapsed across the two visual capture trials to provide an overall *Ownership* *visual capture* score, per participant.

**2.1.1 Main effect: Visual Capture**

To examine the effects of mere visual capture towards subjective ownership of the mannequin body, we compared *Ownership* scores with *Control* scores in the *embodiment questionnaire*. A Wilcoxon signed-rank test revealed a main effect of visual capture, with significantly higher *Ownership* scores compared with *Control* scores (*Z* = -3.26, *p* = .001, *r* = 51).

**2.1.2 Main effect: Tactile Disruption**

In order to determine whether tactile disruption to participants’ own unseen arm would disrupt subjective ownership, we compared *Ownership* scores between *tactile disruption* and *visual capture* conditions. A Wilcoxon signed-rank test revealed a main effect of condition, in which participants showed significantly lower subjective ownership following *tactile disruption* trials (median = -.67) compared with *visual capture* trials (median = .25) (Z = -3.98, *p* < .001, *r* = .63).

**2.1.3 Main effect: Tactile Velocity**

Next, we examined whether tactile velocity had an effect in disrupting the subjective ownership towards the mannequin body within *tactile disruption* trials. A Wilcoxon signed-rank test revealed that there was no significant difference in *Ownership* scores between affective and non-affective tactile disruption trials (*Z* = -.411, *p* = .681, *r* = .06), which suggests that interoceptive affective touch did not disrupt visual capture of ownership to a greater degree than exteroceptive, non-affective touch.

- 1. **Embodiment Questionnaire - Location Sub-component Analysis**

Preliminary analysis showed that there was no effect of trial order across visual capture trials, with a Wilcoxon signed-rank test revealing no significant difference in *Location* scores between visual capture trial 1 vs. trial 2 (*Z* = -.52, *p* = .604). Therefore, *Location* questionnaire scores were collapsed across the two visual capture trials to provide an overall *visual capture* *Location* score, per participant.

**2.2.1 Main effect: Visual Capture**

To examine the effects of mere visual capture towards subjective ownership of the mannequin body, we compared *Location* scores with *Control* scores in the *embodiment questionnaire*. A Wilcoxon signed-rank test revealed a main effect of visual capture, with significantly higher *Location* scores compared with *Control* scores (*Z* = -5.21, *p* < .001, *r* = 82).

**2.2.2 Main effect: Tactile Disruption**

In order to determine whether tactile disruption to participants’ own unseen arm would disrupt subjective location, we compared *Location* scores between *tactile disruption* and *visual capture* conditions. A Wilcoxon signed-rank test revealed a main effect of condition, in which participants showed significantly lower felt location towards the mannequin following *tactile disruption* trials (median = 1.00) compared with *visual capture* trials (median = 2.00) (Z = -3.47, *p* = .001, *r* = .55).

**2.2.3 Main effect: Tactile Velocity**

Next, we examined whether tactile velocity had an effect in disrupting the subjective location that participants felt within *tactile disruption* trials. A Wilcoxon signed-rank test revealed that there was no significant difference in *Location* scores between affective and non-affective tactile disruption trials (*Z* = -.054, *p* = .957, *r* = .01), which suggests that interoceptive affective touch did not disrupt felt location to a greater degree than exteroceptive, non-affective touch.

1. **Results – Combined Samples**
   1. **Subthreshold Eating Disorder Psychopathology - Ownership Sub-component Analysis**

A Spearman’s rank correlation revealed no significant correlation between visual capture *ownership* scores and global EDE-Q scores (*r* = .043, *p* = .71), or any EDE-Q subscale scores (all *ps* > .05).

- 1. **Subthreshold Eating Disorder Psychopathology - Location Sub-component Analysis**

A Spearman’s rank correlation revealed no significant correlation between visual capture *location* scores and global EDE-Q scores (*r* = .038, *p* = .74), or any EDE-Q subscale scores (all *ps* > .05).
